# Supplementary figures and images for: Effects of passages through an insect or a plant on virulence and physiological properties of the fungus Metarhizium robertsii
Source: PeerJ. 2023 Aug 11;11:e15726. doi: 10.7717/peerj.15726 (PMC10424674; doi:10.7717/peerj.15726)

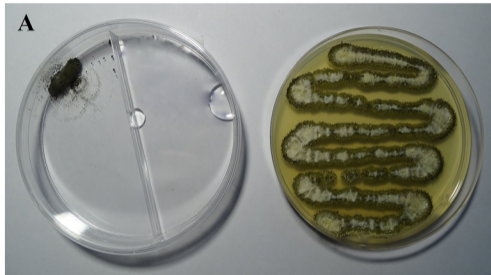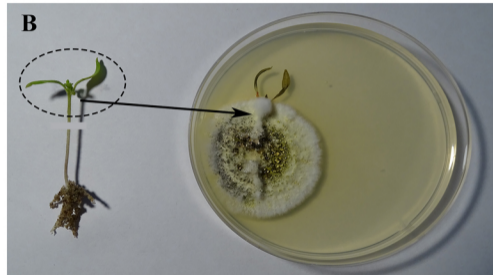

Supplement: Supplemental Information 1 — (А) Reisolate from perished wax moth larvae. (В) Reisolate from tomato seedlings. [file peerj-11-15726-s001.pdf]

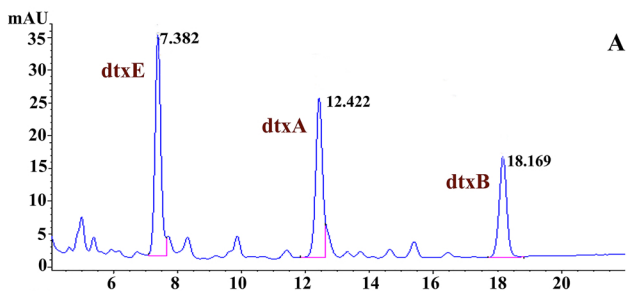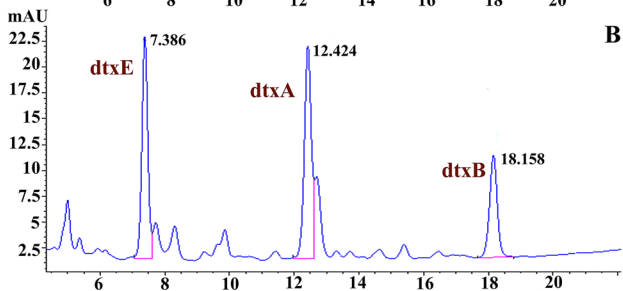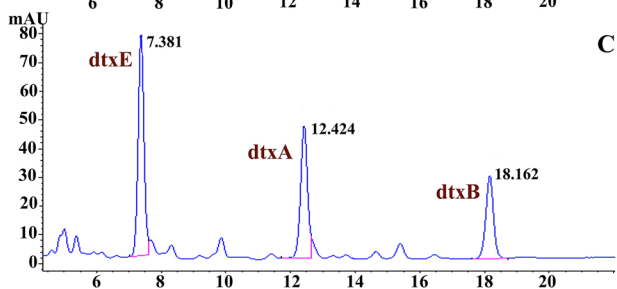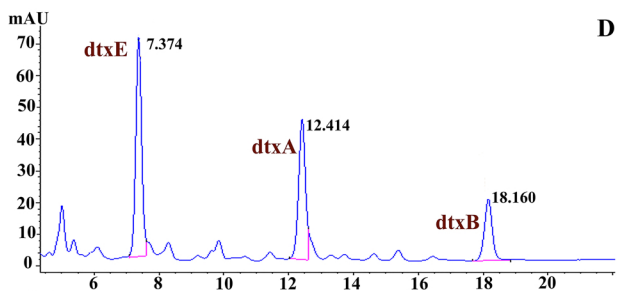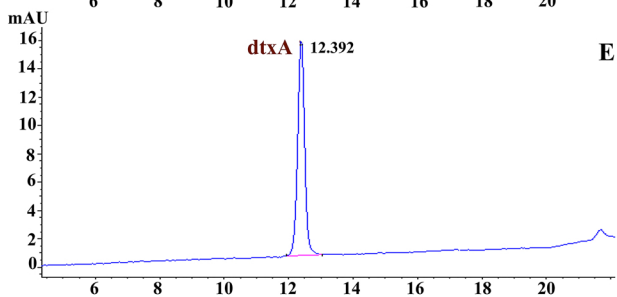

Supplement: Supplemental Information 2 — (A) Parent strain. (B-D) Reisolates after 8 cycles of subculturing on SDAY medium (B) or passaging through tomato (C) and wax moth (D). (Е) Destruxin A standard. [file peerj-11-15726-s002.pdf]

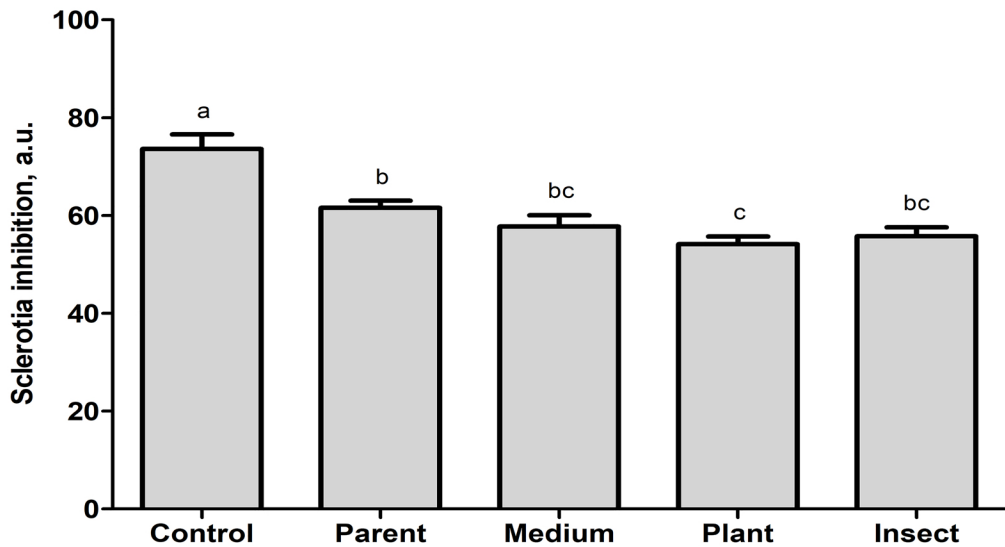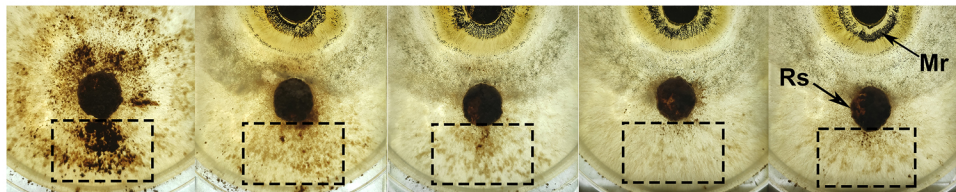

Supplement: Supplemental Information 3 — The two fungi were cocultivated for 20 days. The dashed line indicates borders of the sample zone used for quantitation of the color intensity corresponding to the density of primordia of sclerotia using the ImageJ software (n = 6). Different letters indicate significantly different values (Fisher’s LSD test, p < 0.05). [file peerj-11-15726-s003.pdf]

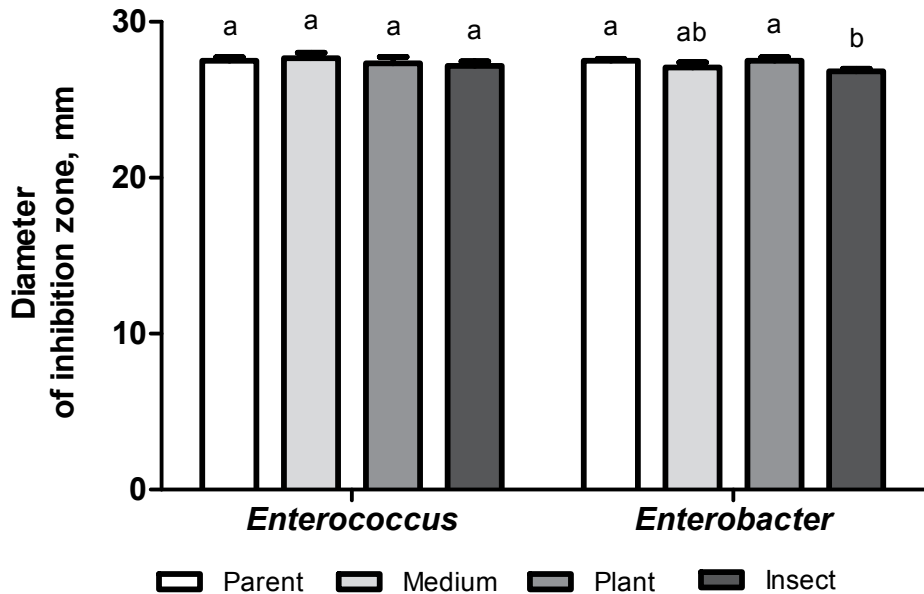

Supplement: Supplemental Information 4 — Growth inhibition was assayed on day 4 as “sterile zone” diameter formed after placing of fungal plugs onto the bacterial culture plated on PDA. Different letters indicate significantly different values (Fisher’s LSD test, p < 0.05). [file peerj-11-15726-s004.pdf]
